# The frequency distribution of reported THC concentrations of legal cannabis flower products increases discontinuously around the 20% THC threshold in Nevada and Washington state

**DOI:** 10.1186/s42238-021-00064-2

**Published:** 2021-03-14

**Authors:** Michael J. Zoorob

**Affiliations:** Department of Government, Harvard University, 1737 Cambridge St, Cambridge, MA 02138 USA

**Keywords:** Cannabis, Laboratory testing, Potency, THC, Lab shopping, McCrary Test

## Abstract

**Background:**

Cannabis laboratory testing reliability is a scientific and policy challenge in US states with legal cannabis. Greater reported THC concentration yields higher prices, and media reports describe a well-known consumer and dispensary preference for flower products containing a minimum 20% THC content—an economically meaningful but biologically arbitrary threshold. This paper examines the frequency distribution of reported THC concentration in legal cannabis flower products in Nevada and Washington state for unusual shifts around the 20% threshold suggestive of potential manipulation of reported THC results.

**Methods:**

THC concentration test results for 142,000 Chemotype 1 flower products from Washington state between June 2014 and May 2017 and 55,000 flower products from Nevada between December 2017 and January 2020 were analyzed for changes in the frequency distribution around the 20% threshold using the McCrary density test. Analyses were performed among all labs in each state, the highest volume lab in Washington, and two labs in Washington which had their licenses suspended for testing irregularities during the study period.

**Results:**

Comparing just above the 20% THC threshold with just below it, the frequency of test results increased by about 43% in Nevada (*z* = 15.6, *p* < 0.001) and by about 17% in Washington (*z* = 11.0, *p* < 0.001). In Washington’s highest volume testing lab, frequency increased by only about 1% (*z* = 0.39, *p* = 0.70), while it increased by about 47% (*z* = 12.7, *p* < 0.001) among the two suspended labs. Subset to those growers which sent products to both sets of labs, frequency of flower products just above the 20% threshold increased by 2% in Washington’s largest lab (*z* = 0.50, *p* = 0.62) and by 52% among the two suspended labs (*z* = 12.8, *p* < 0.001).

**Discussion:**

There is a statistically unusual spike in the frequency of products reporting just higher than 20% THC in both states consistent with economic incentives for products to contain at least 20% THC. This “bunching” of reported THC levels exists among some, but not all, cannabis testing labs, suggesting that laboratory differences (rather than precise manipulation by growers) drive this potential manipulation in reported THC content. These findings elaborate on prior research highlighting unexplained interlaboratory variation in cannabis testing results and highlight ongoing irregularities with legal cannabis testing.

**Conclusion:**

These findings highlight the need for industry oversight and cautions researchers working with reported cannabis THC concentration data, which may be biased by economic incentives to report higher THC.

Medical and adult legal cannabis consumers, and public health analysts, rely on the THC concentration of cannabis products reported by testing laboratories. However, substantively meaningful differences between testing labs in the cannabinoid content of legal cannabis products in Washington state persisted even after adjusting for factors including the product type, strain-name, grower, and testing date (Jikomes and Zoorob 2018), and the labeled dosages of medical cannabis edible products in California and Washington state substantially exceeded, in 60% of cases, the levels obtained by researchers who re-tested them (Vandrey et al. 2015). These discrepancies may be shaped by economic incentives. In legal cannabis markets, products with greater reported THC content sell for higher prices (Smart et al. 2017), and press reports describe a substantial benefit to flower products containing just higher than 20% THC. In Nevada, “The standard is 20 percent and higher. No one wants a THC level under that” (Gentry 2019). In Washington, “… [M]any stores won’t even bother looking at anything that tests under 20 percent” (Coughlin-Bogue 2016). Given these store and consumer preferences, growers may have an incentive to produce cannabis batches with labeled THC levels greater than 20% because stores pay more for such products (Downs 2019). In turn and aided by the latitude afforded by natural variability in cannabis test results and testing processes, labs may have an economic incentive to report THC concentrations of just above 20% to retain business with growers (Gentry 2019). Growers freely choose which labs test their products, and industry stakeholders suggest that at least some growers exercise this discretion by sending products to labs based on the expected THC results (so-called “lab-shopping”). One testing lab executive explained that “We certainly have clients [growers] that are comparing our cannabinoid results against other labs and telling us straight up that they’re making decisions based on cannabinoid results” (Downs 2019).

The 20% THC threshold is economically meaningful, but biologically arbitrary. Because of biological variability and assay variability, the frequency distribution of reported THC concentrations would be expected to be smooth around 20% threshold. Hence, this paper tests two hypotheses. First, the null hypothesis of no manipulation predicts that there will be approximately as many products testing just below as just above 20% THC. Second, the alternative hypothesis that economic incentives influence reported THC concentrations predicts that there would be an unusual “bunching” of products just above the 20% THC threshold. To adjudicate between these hypotheses, this paper examines the frequency distributions of reported THC concentration in legal cannabis flower products in Nevada and Washington state. A McCrary density test is used to examine whether the frequency of products shifts discontinuously around the 20% threshold, suggestive of potential manipulation of reported THC results.

## Methods

The analyses used testing data for legal flower products tested in Washington between June 2014 and May 2017 (Jikomes and Zoorob 2018) and Nevada between December 2017 and January 2020 (obtained via public records request to the Nevada Department of Taxation). These states and years were chosen due to data availability; requests were also submitted to other states with legal cannabis markets but did not produce responsive records.

Both Washington and Nevada require representative samples of all plants used for flower products to be tested for potency (specifically the cannabinoids THC, THCA, CBD, and CBDA) and require some form of lab accreditation. Though each states’ regulations provide some guidance as to acceptable methodologies, neither state requires the use of specific analytical methodologies for potency testing. In Nevada, state regulations (NAC 453D.764) require the use of “analytical methods approved by the Department [of Taxation].” In Washington (WAC 314-55-102), “Regardless of analytical equipment or methodology, certified labs must accurately measure and report the acidic (THCA and CBDA) and neutral (THC and CBD) forms of the cannabinoids.”

Flower products were identified in both states from fields in the data distinguishing cannabis product types; in Nevada, data were subset to those tests whose Category field was “Marijuana Flowers/Buds” and, in Washington, those whose Inventory Type was “Flower Lot.” THC reported on product labels was the total potential THC (or maximum THC), legally defined as 0.877*THCA + THC (see NAC 453D.100 and WAC 314-55-109). Contrary to scientific literature (e.g., Hädener et al. 2019), this definition assumes the conversion of THCA molecules to THC molecules when heated is perfectly efficient. All analyses used the total potential THC (hereon THC). Following other research (Stith et al. 2019; Stith et al. 2020), tests reporting higher than 35% THC (biologically dubious levels) are dropped, though this does not change results. The main analytic dataset included 142,847 Chemotype-1 (those with a THC:CBD ratio exceeding 5) products in Washington and 55,523 in Nevada.

The laboratory data were tested for unusual “bunching” (that is, a discontinuous change in the frequency) of labeled THC content. A McCrary test was used to assess whether there was an unusually high frequency of products containing just above the 20% THC threshold (McCrary 2008). The McCrary test is a common statistical technique which examines the null hypothesis that the frequency of observations (or, formally, a variable’s “probability density function”) is continuous on both sides of a threshold value; it essentially applies a regression discontinuity design analysis to the histogram of a variable and is often used as a diagnostic technique in regression discontinuity designs to assess the likelihood that units are influencing their treatment assignment (McCrary 2008). Intuitively, a McCrary test assesses whether there are about the same number of observations above a threshold value as below that threshold, as would be expected by chance, or whether there is statistically unusual “bunching” of results on one side of the threshold. The McCrary test also has been used to suggest the presence of manipulation in a variety of settings where actors have an incentive to misreport in order to secure economic benefits, including procurement contracts (Palguta and Pertold 2017), agricultural production (Zhang et al. 2019), and endowment returns (Almond and Xia 2017). Analyses use the *DCdensity* function in the *R* (R Core Team 2020) programming language’s *rdd* library (Dimmery 2016) with default bandwidth selection (McCrary 2008). Replication materials (code and data) reproducing all numerical results and figures are freely available for download on the Harvard Dataverse repository (see data availability statement).

If growers were precisely manipulating the THC content of their plants to avoid producing samples containing just below 20% THC, then sorting patterns would likely appear among all labs. However, sorting patterns among only some labs provide evidence consistent with variation driven at the laboratory level. This was explored with the Washington data, which identifies laboratories. Two of the six biggest laboratories had their licenses suspended for testing irregularities during the data collection period (Jikomes and Zoorob 2018). The McCrary test was repeated separately for (1) the two suspended labs (*n* = 35,170) and (2) the lab with the highest number of tests (*n* = 39,981). To gain further leverage on whether labs or growers drive the changes in frequency, this analysis was repeated again using only data from growers who sent their products to both the biggest lab and the two suspended labs during the study period. Tests from those growers who sent their products only to one set of labs are omitted, resulting in 29,336 tests from the two suspended labs and 21,964 tests from the biggest lab in the analysis.

## Results

**Fig. 1:**
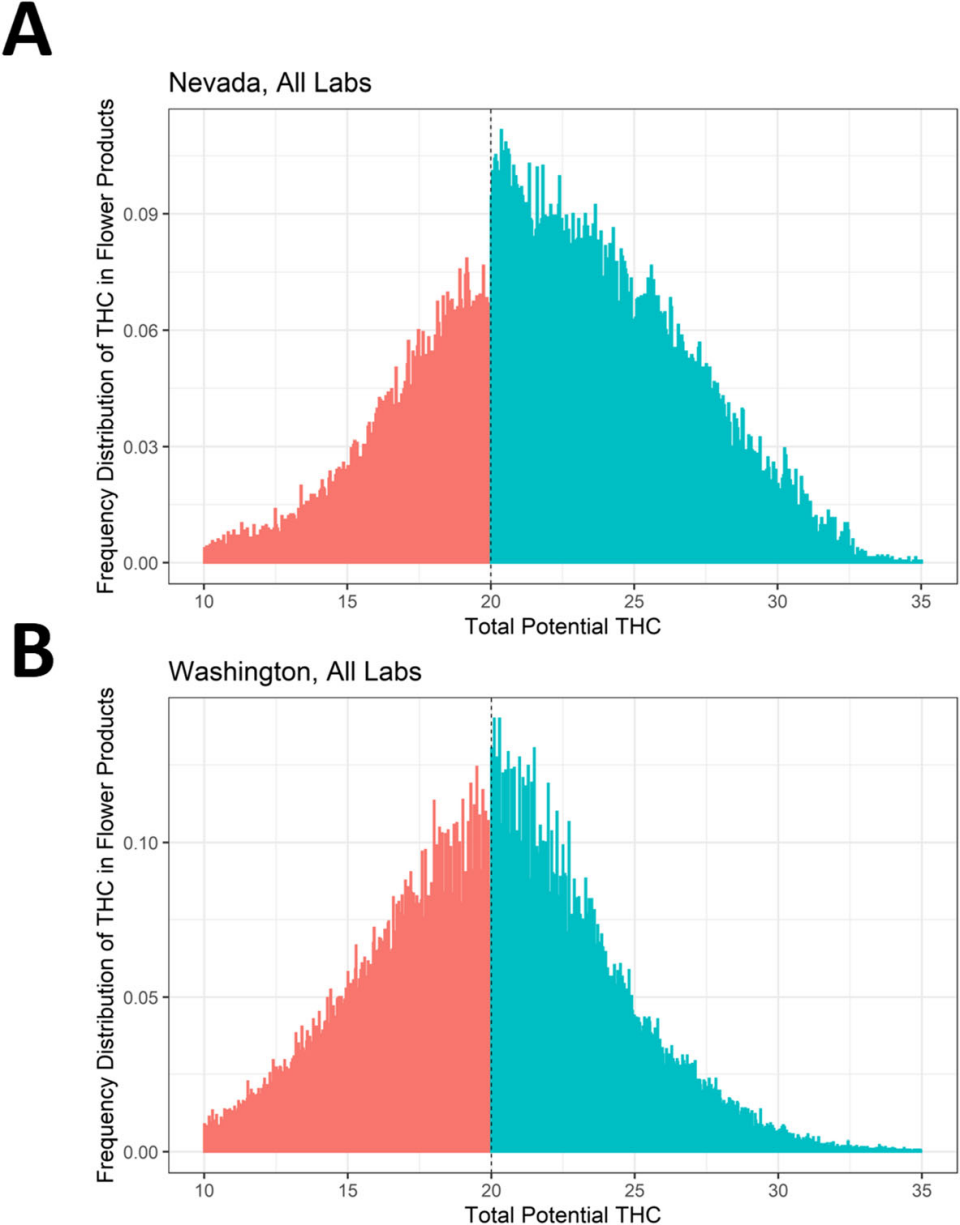
Frequency of reported THC content in flower products around the 20% threshold in Nevada and Washington. This figure consists of two plots showing histograms of the frequency of reported THC, with bins to the left of the 20% threshold shaded red and bins to the right of the 20% threshold shaded blue. The top plot (panel **a**) shows data from all labs in Nevada, and the bottom (panel **b**) shows all labs in Washington. Both states show a spike in the frequency of products just above the 20% threshold, though the increase is larger in Nevada. Plots are left-truncated at 10% for presentational purposes. Histogram bins were generated via the *DCdensity* function

Figure 1 shows the distributions of labeled THC in flower products. In Nevada, pooling data from all labs, there is strong visual and statistical evidence of “bunching” around the 20% threshold, with the frequency of products reporting just above 20% THC sharply exceeding the frequency of products reporting just below 20% THC (Fig. 1, top left) and a log difference in frequency heights at the threshold of 43% (*z* = 15.6, *p* < 0.001). Testing data from Washington shows similar, though somewhat less dramatic, evidence of bunching, with a log difference in frequency heights of about 17% (Fig. 1, top right; *z* =11.0; *p* < 0.001).

**Fig. 2:**
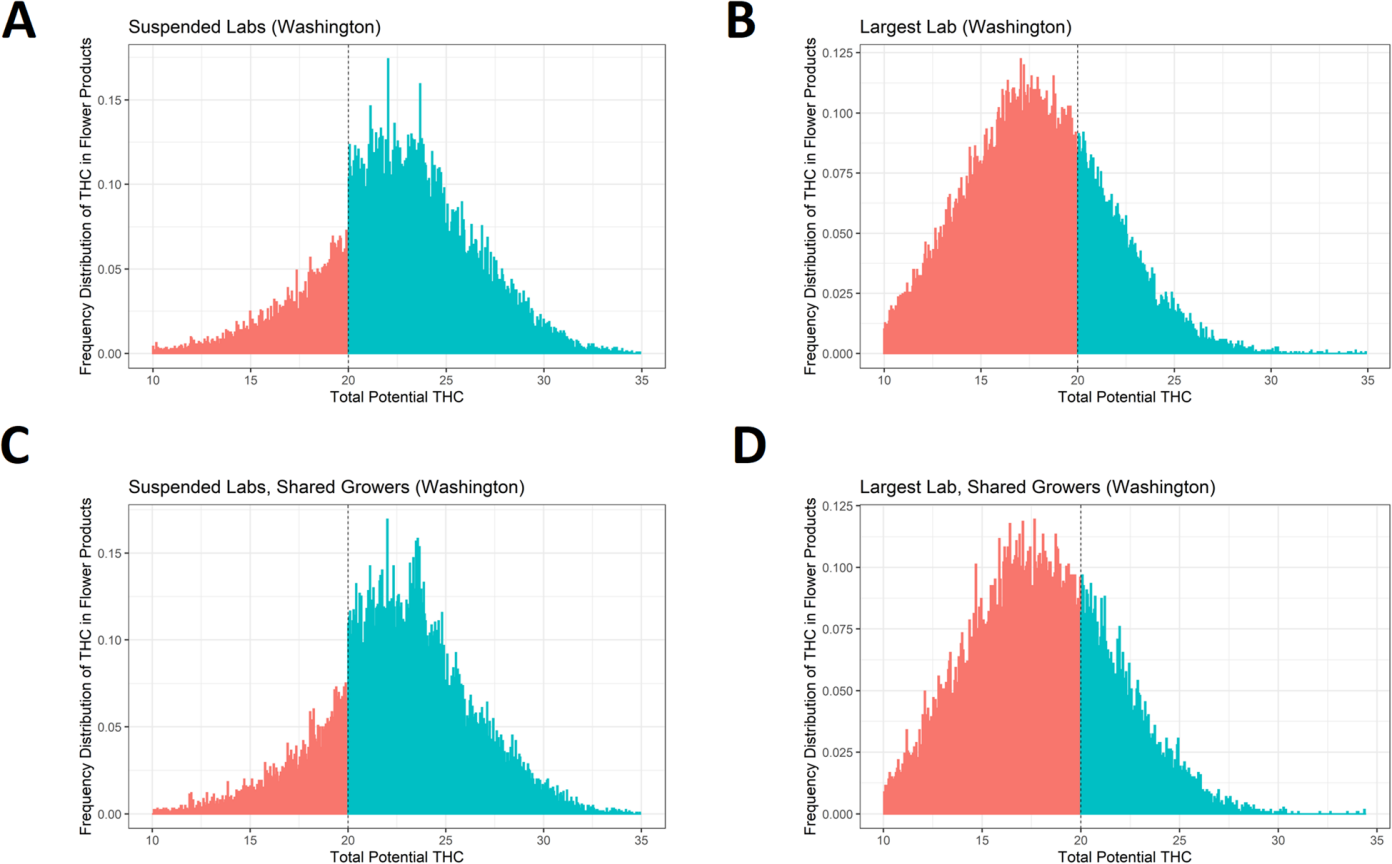
Frequency of reported THC content around the 20% threshold among subsets of labs. This figure consists of four plots showing histograms of the frequency of reported THC, with bins to the left of the 20% threshold shaded red and bins to the right of the 20% threshold shaded blue. Plots are left-truncated at 10% for presentational purposes. The top left plot (panel **a**) shows data from two labs in Washington state which had their licenses suspended (“suspended labs”), and the top right plot (panel **b**) shows data from the lab in Washington which tested the most products during the study period (“largest lab”). The bottom row also plots histograms from the suspended labs (left; panel **c**) and the largest lab (right; panel **d**) but only using testing data from those growers which sent products to both sets of labs. While the two suspended labs show sharp increases in frequency at the 20% threshold (panels **a** and **c**), the frequency distribution for the largest lab is smooth across the 20% THC threshold (panels **b** and **d**). Histogram bins were generated via the *DCdensity* function

Among suspended labs (Fig. 2, panel a), there are unusually fewer products testing just below 20% THC and unexpectedly more products testing above 20% THC, with a 47% increase in frequency at the 20% threshold (*z* = 12.5, *p* < 0.001). However, among the largest lab (Fig. 2, panel b), there is only a 1% change in the frequency of products around 20%, statistically indistinguishable from no change (*z* = 0.39, *p* = 0.70). Among this subset of tests from overlapping growers, a discontinuous increase in frequency of observations at the 20% threshold is again evident among the suspended labs, with a 52% increase in frequency (Fig. 2c, *z* = 12.8, *p* < 0.001), but not among the biggest lab, with a 2% increase that was not statistically distinguishable from zero (Fig. 2d, *z* = 0.50, *p* = 0.62).

**Fig. 3:**
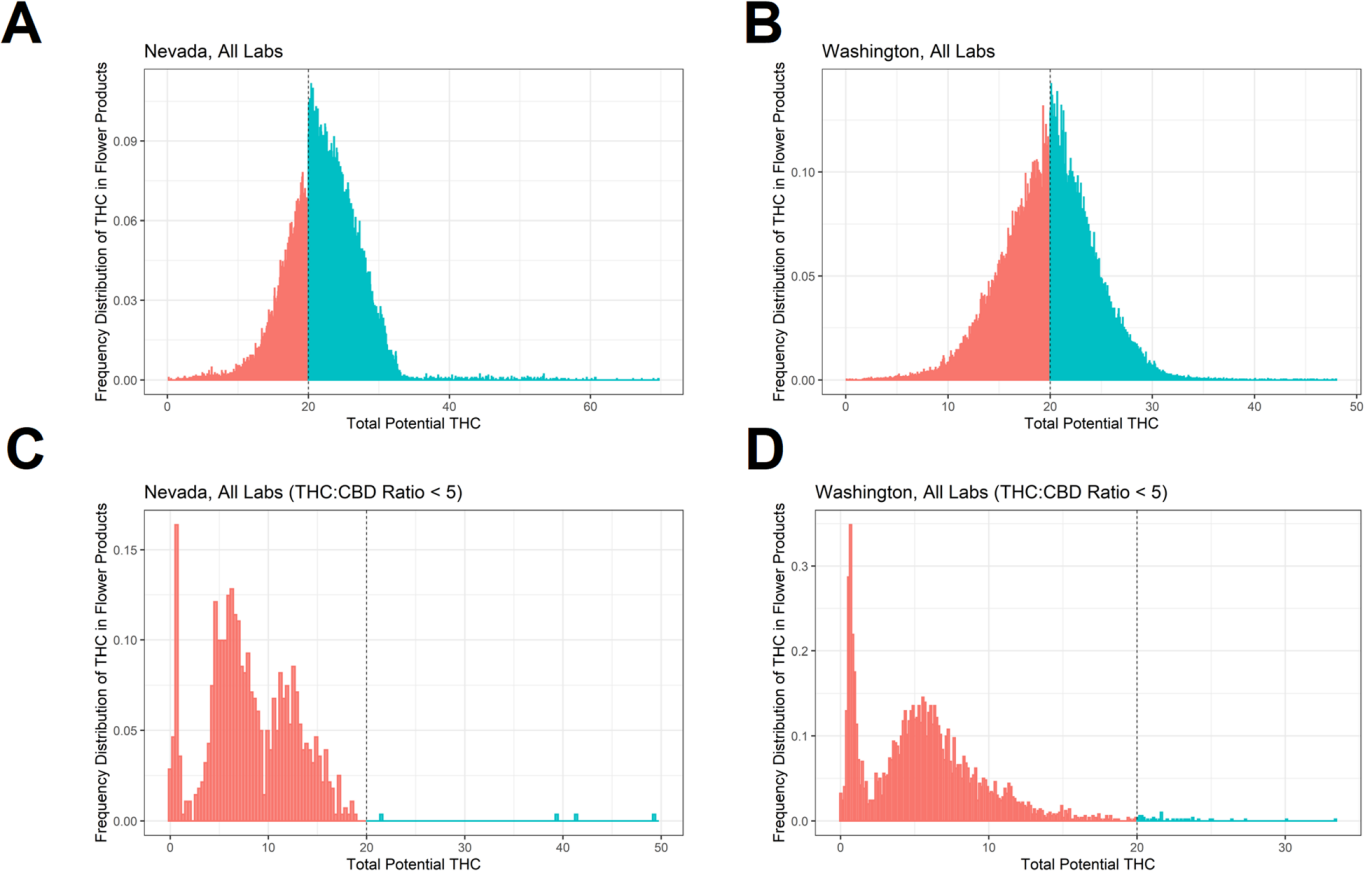
Sensitivity tests including products above 35% THC and products with THC:CBD ratios of less than 5. This figure consists of four plots showing histograms of the frequency of reported THC, with bins to the left of the 20% threshold shaded red and bins to the right of the 20% threshold shaded blue. The top row is similar to the top row of Fig. 1, except that products above 35% THC are included, and there is no left-truncation of plots at 10% THC. The top left plot (panel **a**) shows data from Nevada and the top right plot (panel **b**) shows data from Washington state; both panels show a spike in reported THC concentrations around the 20% threshold. The bottom row shows the THC frequencies for flower products with THC:CBD ratios of less than 5 (i.e., mixed or predominantly CBD products) in Nevada (panel **c**) and Washington (panel **d**). Histogram bins were generated via the *DCdensity* function. In both states, there are few products with such THC:CBD ratios around the 20% THC threshold

In sensitivity tests, histograms as in Fig. 1 are shown while including those test results reporting concentrations in excess of 35% THC (Fig. 3a, b). Similar increases in frequency at the 20% threshold reported in Fig. 1 are evident in both states and the McCrary test results are not substantially changed by including these products (the McCrary test analyzes only those data within a narrow neighborhood around the threshold value, which is 20% THC). The frequency distributions of THC content in those flower products with THC:CBD concentrations of less than 5 (i.e., hemp products and “mixed” THC-CBD products) are plotted for Nevada and Washington (Fig. 3c, d, respectively). There are very few such products with THC concentrations near 20%, and there is little visual evidence of any substantial changes in test frequency around the 20% THC threshold.

## Discussion

In both Nevada and Washington, an unusually high frequency of flower products report THC concentrations just higher than 20% THC (and an unusually small frequency report just below 20% THC). This discontinuity is evident for some, but not all, labs; and it persists even when examining products from the same growers. This suggests that potential manipulation by some laboratories, rather than some growers, drives the result. Of course, some growers may also manipulate the samples they send to laboratories for testing in order to obtain higher THC results; however, sorting around the 20% THC threshold appears to be better explained by laboratory-level differences. Building on other scholarship identifying discrepancies in labeled THC content (Vandrey et al. 2015) and variation between cannabis testing labs in reported THC concentrations in Washington state (Jikomes and Zoorob 2018), as well as analyses by industry analysts described in blogs and media reports suggesting “lab-shopping” (Gentry 2019; Downs 2019), this study provides a direct statistical test suggestive of potential manipulation in THC concentrations and applies it to cannabis testing data from two states. Cannabis analysts have raised the issue of “lab shopping” in legal cannabis markets, where growers send products to different laboratories to obtain higher THC results (Gentry 2019). This study also has several limitations which provide opportunities for future research. First, the analyses are limited to flower products. While manipulation in reported THC concentrations in other types of cannabis products (e.g., concentrates, edibles) is plausible, there is not a sharp threshold akin to the 20% THC threshold for flower products to use for similar analyses, so researchers may need alternative techniques to assess potential manipulation in reported THC concentration. Second, the analyses are limited to markets in just two states and for a limited time frame, making comparisons between cannabis testing regulatory environments and changes over time difficult to determine. To the extent that such data limitations can be overcome, future research identifying policy and regulatory changes that appear to diminish potential manipulation of reported THC would be most welcome. Finally, the approach is limited in that the McCrary test method can only provide statistical evidence that is suggestive of manipulation in THC concentration; it cannot directly demonstrate improper behavior.

These findings underscore the need for oversight of legal cannabis laboratories to ensure data reliability and suggest that the McCrary test provides a straightforward circumstantial test of one form of potential laboratory manipulation. In the meantime, researchers and consumers need to tread cautiously when interpreting cannabis lab testing data to make inferences about THC concentration.

## Data Availability

Code and data reproducing all figures and numerical results are freely accessible from the Harvard Dataverse at the following DOI: 10.7910/DVN/GNFVBS.

## Abbreviations

THC: Tetrahydrocannabinol
THCA: Tetrahydrocannabinolic acid
CBD: Cannabidiol
NAC: Nevada Administrative Code
WAC: Washington Administrative Code
